# The inhibition of circular RNA circNOLC1 by propofol/STAT3 attenuates breast cancer stem cells function via miR-365a-3p/STAT3 signaling

**DOI:** 10.1186/s12967-021-03133-5

**Published:** 2021-11-17

**Authors:** Yi-Ping Liu, Jin-Yu Heng, Xin-Yu Zhao, En-You Li

**Affiliations:** 1 Department of Anesthesiology, The First Affiliated Hospital of Harbin Medical University, No. 23 Youzheng Street, Harbin, Heilongjiang Province China; 2 The First Affiliated Hospital of Harbin Medical University, Harbin, Heilongjiang Province China

**Keywords:** Breast cancer, Progression, CSCs, circNOLC1, miR-365a-3p, STAT3, Propofol

## Abstract

**Background:**

Breast cancer remains one of the most dreadful female malignancies globally, in which cancer stem cells (CSCs) play crucial functions. Circular RNAs have drawn great attention in cancer research area and propofol is a widely applied intravenous anesthetic agent. Methods: In the current study, we explored the function of circular RNA nucleolar and coiled-body phosphoprotein 1 (circNOLC1) in CSCs of breast cancer and the inhibitory impact of propofol on circNOLC1.

**Results:**

The expression of circNOLC1 was induced in breast cancer tissues compared with the non-tumor tissues. The silencing of circNOLC1 was able to repress the viability of breast cancer cells. Meanwhile, the numbers of colony formation were suppressed by circNOLC1 knockdown in breast cancer cells. The inhibition of circNOLC1 reduced the invasion and migration ability of breast cancer cells. The mRNA and protein levels of E-cadherin were enhanced but Vimentin levels were reduced by the silencing of circNOLC1. The repression of circNOLC1 decreased the side population (SP) ratio in breast cancer cells. Meanwhile, the sphere formation ability of breast cancer cells was attenuated by the silencing of circNOLC1. The levels of ATP-binding cassette (ABC) superfamily G member 2 (ABCG2), c-Myc, B cell-specific Moloney murine leukemia virus integration site 1 (Bmi1), and SRY-box transcription factor 2 (Sox2) were repressed by the depletion of circNOLC1 in the cells. Regarding to the mechanism, circNOLC1 functioned as a competing endogenous RNAs (ceRNAs) for microRNA-365a-3p (miR-365a-3p) and the inhibition of miR-365a-3p rescued circNOLC1 depletion-repressed proliferation and cancer stem cell activity of breast cancer. MiR-365a-3p targeted signal transducer and activator of transcription 3 (STAT3) in breast cancer cells and circNOLC1 enhanced STAT3 expression by sponging miR-365a-3p. The overexpression of STAT3 could reverse miR-365a-3p or circNOLC1 depletion-inhibited proliferation and cancer stem cell properties of breast cancer. Interestingly, the expression of circNOLC1 and STAT3 was repressed by the treatment of propofol. The enrichment of STAT3 on circNOLC1 promoter was inhibited by propofol. The expression of circNOLC1 was suppressed by the silencing of STAT3 in the cells. The inhibition of circNOLC1 expression by propofol was rescued under the co-treatment of STAT3 overexpression. The overexpression of circNOLC1 rescued propofol-attenuated proliferation and cancer stem cell functions in vitro and in vivo.

**Conclusions:**

Thus, we concluded that circNOLC1 contributes to CSCs properties and progression of breast cancer by targeting miR-365a-3p /STAT3 axis and propofol inhibited circNOLC1 by repressing STAT3 in a feedback mechanism.

## Background

Breast cancer (BC) remains one of the most dreadful female malignancies globally, despite the development of targeted therapy, radiotherapy and immunotherapy, due to the acquired therapeutic resistance and cancer stem cells (CSCs) [1]. BC has been recognized as a malignancy that displays tumor heterogeneity of complex cell types [2]. Breast cancer stem cells (BCSCs), the subpopulation of breast cancer cells that exhibit self-renewal and differentiation ability, were suggested as an important participate of tumor heterogeneity, and participated in the carcinogenesis, progression, and therapeutic resistance of breast cancer [3]. For example, the portion of BCSCs was remarkably elevated in breast tumors that resistant to clinical therapies, owing to their survival advantages over DNA damage induced by chemo-drugs [4]. Besides, BCSCs were able to form tumor blood vessels via differentiating into smooth muscle-like cells and endothelial cells [5]. It is suggested that STAT3 signaling pathway played an important role in the self-renewal of BCSCs and functioned as potential therapeutic targets [6, 7]. Deciphering the regulatory mechanisms of BCSCs and targeting this special subtype of cancer cells were thus regarded as promising approach for BC therapy.

Circular RNA (circRNA), as a new type of non-coding RNA, have drawn great attention in cancer research area [8]. Owing to the special covalently closed structure, circRNAs are more stable in physical conditions and resistant to degradation caused by ribonuclease R, therefore exhibit higher levels in various cancer tissues, and are regarded as ideal diagnostic biomarkers and therapeutic targets [9–11]. A great number of circRNAs have been indicated to participate in development of BC, through transcriptionally regulate the levels of microRNAs (miRNAs) and mRNAs [12]. For example, circ-DNMT1 interacted with p53 to promote its nuclear translocation and induced autophagy of BC cells [13]. Zeng et al. reported a circ-ANKS1B highly expressed in BC and closely related to advanced clinical stage and metastasis to lymph node [14]. CircNOLC1 was recently discovered to be overexpressed in prostate cancer by a microarray analysis, and affect prostate cancer cell proliferation and metastasis [15]. As critical cancer regulators, miRNAs participate in various biological processes during carcinogenesis via suppressing targeted mRNA translation [16]. MiR-365a-3p is indicated as a suppressor in several cancers, including pancreatic cancer, colorectal cancer, gastric cancer and so on [17–19]. However, its role in BC is unclear yet.

Propofol is a widely applied intravenous anesthetic agent, that was also implied to affect the progression of breast cancer. In the mice model of BC surgery, administration of propofol could effectively suppress the pulmonary metastasis of BC cells [20]. Noteworthy, Zhang and colleagues suggested that propofol reduced the self-renewal ability of BCSCs, possibly through regulating the PD-L1 [21]. In this work, we aimed to determine the role of circNOLC1 during the suppression of BCSCs by propofol, and discovered a miR-365a-3p/STAT3 regulatory axis involved in this process.

## Methods

### Clinical specimen

Human breast cancer tissues (n = 30) and the adjacent nontumor tissues (n = 30) were obtained from our hospital BC patients with who hospitalized at the our hospital from 2016 to 2020. The tissues were stored at liquid nitrogen. For the RNA extraction, the tissues were treated with TRIzol reagent. Experiments in this study were ratified by the Clinical Ethnic Committee of First Affiliated Hospital of Harbin Medical University. All patients have signed the informed content before the start of experiments.

### Cell lines and materials

Human BC cell lines MDA-MB-231 and MDA-MB-468 were purchased from the Cell Bank of the Chinese Academy of Sciences (China), cultured in high glucose DMEM (Hyclone, USA) supplemented with 10% fetal bovine serum (FBS, Gibco, USA) and 1% penicillin/streptomycin (Sigma, USA). Cells were placed in a 37 °C incubator filled with 5% CO_2_. Propofol was obtained from Sigma and used at a dose of 10 µM for cellular experiments according to the previous report [22]. ShcircNOLC1, siSTAT3, circNOLC1 overexpressing vectors (pcD-ciR-circNOLC1), STAT3 overexpressing vectors (pcDNA-STAT3), miR-365a-3p mimics and inhibitors, and the negative controls (NC) were synthesized by Geenseed (China). Cell transfection of the oligonucleotides (50 μM) were performed by using Lipofectamine 2000 reagent (Invitrogen).

### Colony formation

BC cells were seeded in a 6-well plate after treatment, and incubated for 12 days to form visible colonies. The colonies were then dyed with 0.5% crystal violate (Beyotime, China) in methanol for 20 min in dark. The images were captured by digital camera (Olympus, Japan).

### Cell viability

The in vitro growth of BC cells was determined by cell counting 8 (CCK-8) kit (Thermo) in accordance with manufacturer’s description. Briefly, 5000 cells were placed into each well of 96-well plates and incubated for 24, 48, 72 and 96 h, respectively. CCK-8 solution was then added into each well to hatch for another 1 h. Absorbance at 450 nm was measured by a microplate reader (Thermo).

### Cell migration and invasion

BC cells (2 × 10^3^) cells were seeded in the upper chamber of the transwell insert (Corning, USA) with serum-free medium. Complete medium was added into the lower chambers. After incubation for 48 h, cells penetrated through the upper chambers were stained with 0.5% crystal violet and photographed.

For wound healing experiment, cells were seeded and incubated to form monolayers, followed by scratching with sterile 200 μl pipette tip. The cells were then incubated in medium containing 0.1% FBS. The images of the scratches were captured at 0 and 24 h.

### Western blotting

Proteins extracted from BC cells using a RIPA lysis buffer containing mixture of protease inhibitors were separated by SDS-PAGE electrophoresis, shifted to NC membranes, blocked with 5% non-fat milk for 2 h at room temperature. The blots were hatched with E-cadherin, Vimentin, ABCG2, C-Myc, Bim21, Sox2, STAT3, SLC7A11, β-actin antibodies (1:2000, Abcam, USA) at 4 ℃ for one night. Subsequently, the protein bands were visualized by incubation with HRP-conjugated secondary antibodies (Abcam) and the ECL agents (Sigma).

### RNA extraction and quantification

Total RNAs of tissues and BC cells were extracted via TRIzol reagent (Invitrogen) and quantified. A total of 1 µg RNA was reversely transcribed to cDNA by using First Strand Reverse Transcription kit (Thermo), followed by real-time PCR analysis with SYBR Green qPCR Master Mix (Thermo). Relative gene expression was normalized to internal control GAPDH or U6 [23]. Relative quantification of circRNA, miRNA and mRNA expression was compared to internal control and analyzed using the 2^−ΔΔCT^ method. Primer sequences are as following:

circNOLC1: forward primer 5′-TGAGCCACCAAAGAACCAGA-3′, reverse primer 5′- AACTTTCGCTCTGGGACCTT-3′;

miR-365a-3p: forward primer 5′- TGCGGTAATGCCCCTAAAAA-3′, reverse primer 5′- TGCAAGAGCAATAAGGATT-3′;

STAT3: forward primer 5′- CAGCAGCTTGACACACGGTA-3′, reverse primer 5′- AAACACCAAAGTGGCATGTGA-3′;

β-actin: forward primer 5′-CAGAGCAAGAGAGGCATCC-3′, reverse primer 5′- CTGGGGTGTTGAAGGTC-3′;

U6: forward primer 5′-CTCGCTTCGGCAGCACA-3′, reverse primer 5′-CTCGCTTCGGCAGCACA-3′.

### Sphere formation

BC cells were trypsinized, suspended in serum-free DMEM/F12 medium (Hyclone), and planted into each well (500 cells) of an ultra-low-attachment 24-well plate (Corning). The medium contains B27 (Thermo), epidermal growth factor (EGF, Thermo), basic fibroblast growth factor (bFGF, Thermo) and methylcellulose (Thermo). Ten days later, the formed spheres were captured and counted by using a microscope (Leica, Germany).

### Luciferase reporter gene assay

The bioinformatics analysis of miR-365a-3p, circNOLC1, and STAT3 was performed in ENCORI database. The wild type and mutated sequences of circNOLC1 and STAT3 were cloned into pmirGLO vectors (QiaGene, Germany) and co-transfected into BC cells along with miR-365a-3p mimics or NC. After 48-h transfection, cells were digested and homogenized. The luciferase activity was evaluated by using the dual-Luciferase reporter assay system of Promega in accordance with manufacturer’s description.

### Pull-down assay

The RNA pull-down experiment with biotin-labelled circNOLC1 probe (Gene Pharma, China) was conducted to determine the interaction between circNOLC1 and miR-365a-3p using a Pierce Magnetic RNA–Protein Pull-Down Kit (Thermo) following the manufacturer’s protocol.

### In vivo study

Female Balb/c mice (4–6 weeks old) were obtained from Vital River Laboratory. The experimental protocol was authorized by First Affiliated Hospital of Harbin Medical University. MDA-MB-231 cells (5 × 10^5^ cells per mouse) transfected with circNOLC1 overexpressing vectors or the control vectors were subcutaneously injected into right-side back. Propofol (50 mg/kg) was intraperitoneally administrated to mice every day when tumor size approached 100 mm^3^. Tumors were isolated, weighted, subjected to paraffin embedding and immunohistochemical (IHC) staining with Ki-67 antibody (Abcam).

### Statistics

The measured data were shown as mean ± S.D of three independent repeats, and analyzed with Student’s *t*-test or two-way-ANOVA using the SPSS software. The significance threshold (*p*) was 0.05.

## Results

### The inhibition of circNOLC1 represses breast cancer proliferation in vitro

**Fig. 1:**
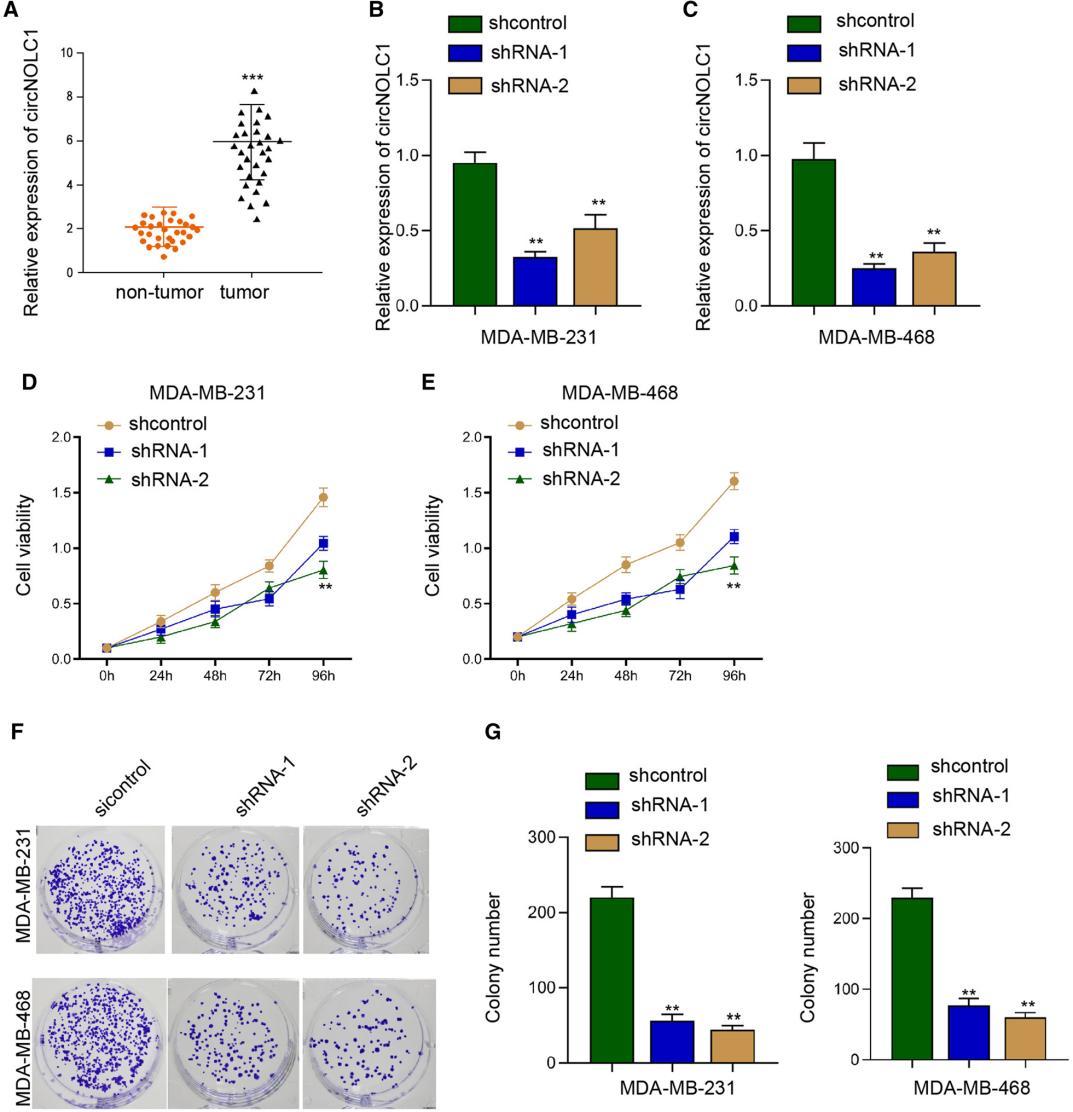
The inhibition of circNOLC1 represses breast cancer proliferation in vitro. **A** The levels of circNOLC1 were examined by qPCR in clinical breast cancer tissues (n = 30). **B**–**G** The MDA-MB-231 and MDA-MB-231 cells were treated with circNOLC1 shRNAs. **B** and **C** The levels of circNOLC1 were validated by qPCR. **D** and **E** The cell viability was detected by CCK-8 assays. **F** and **G** The colony numbers of the cells were measured by colony formation assays. **P < 0.01, ***P < 0.001

To confirm the relationship of circNOLC1 with breast cancer, we examined the levels of circNOLC1 in clinical breast cancer samples. We observed that the expression of circNOLC1 was induced in breast cancer tissues compared with the non-tumor tissues (Fig. 1A). The inhibition efficiency of circNOLC1 shRNAs was validated in MDA-MB-231 and MDA-MB-468 cells (Fig. 1B and C). The silencing of circNOLC1 was able to repress the viability of MDA-MB-231 and MDA-MB-468 cells (Fig. 1D and E)). Meanwhile, the numbers of colony formation were suppressed by circNOLC1 knockdown in MDA-MB-231 and MDA-MB-468 cells (Fig. 1F and G), indicating that the inhibition of circNOLC1 represses breast cancer proliferation in vitro*.*

### The inhibition of circNOLC1 inhibits breast cancer migration and epithelial-mesenchymal transition in vitro

**Fig. 2:**
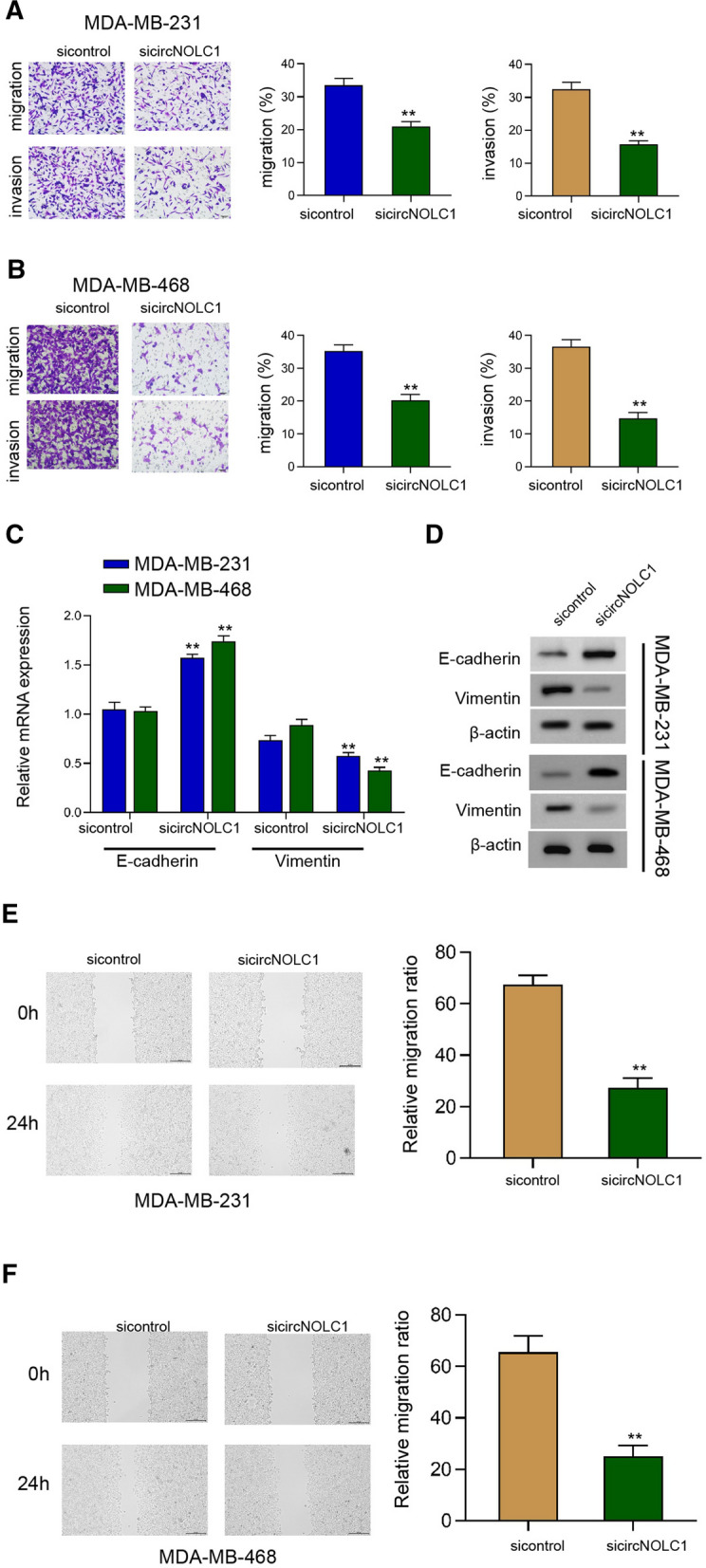
The inhibition of circNOLC1 inhibits breast cancer migration and epithelial-mesenchymal transition in vitro. **A**–**F** The MDA-MB-231 and MDA-MB-468 cells were treated with circNOLC1 shRNAs. **A** and **B** The invasion and migration were measured by transwell assays. **C** and **D** The mRNA and protein levels of E-cadherin and Vimentin were determined by qPCR and Western blot analysis, respectively. **E** and **F** The migration was analyzed by wound healing assays. **P < 0.01

Then, we assessed the role of circNOLC1 in migration and epithelial-mesenchymal transition (EMT). The repression of circNOLC1 was able to reduce the invasion and migration ability of MDA-MB-231 and MDA-MB-468 cells (Fig. 2A and B). The mRNA and protein levels of E-cadherin were enhanced but Vimentin levels were reduced by the silencing of circNOLC1 in MDA-MB-231 and MDA-MB-468 cells (Fig. 2C and D). In addition, the wound healing capability of MDA-MB-231 and MDA-MB-468 cells was inhibited by the depletion of circNOLC1 (Fig. 2E and F), indicating that the inhibition of circNOLC1 inhibits breast cancer migration and EMT in vitro.

### The inhibition of circNOLC1 attenuates cancer stem cell activity of breast cancer

**Fig. 3:**
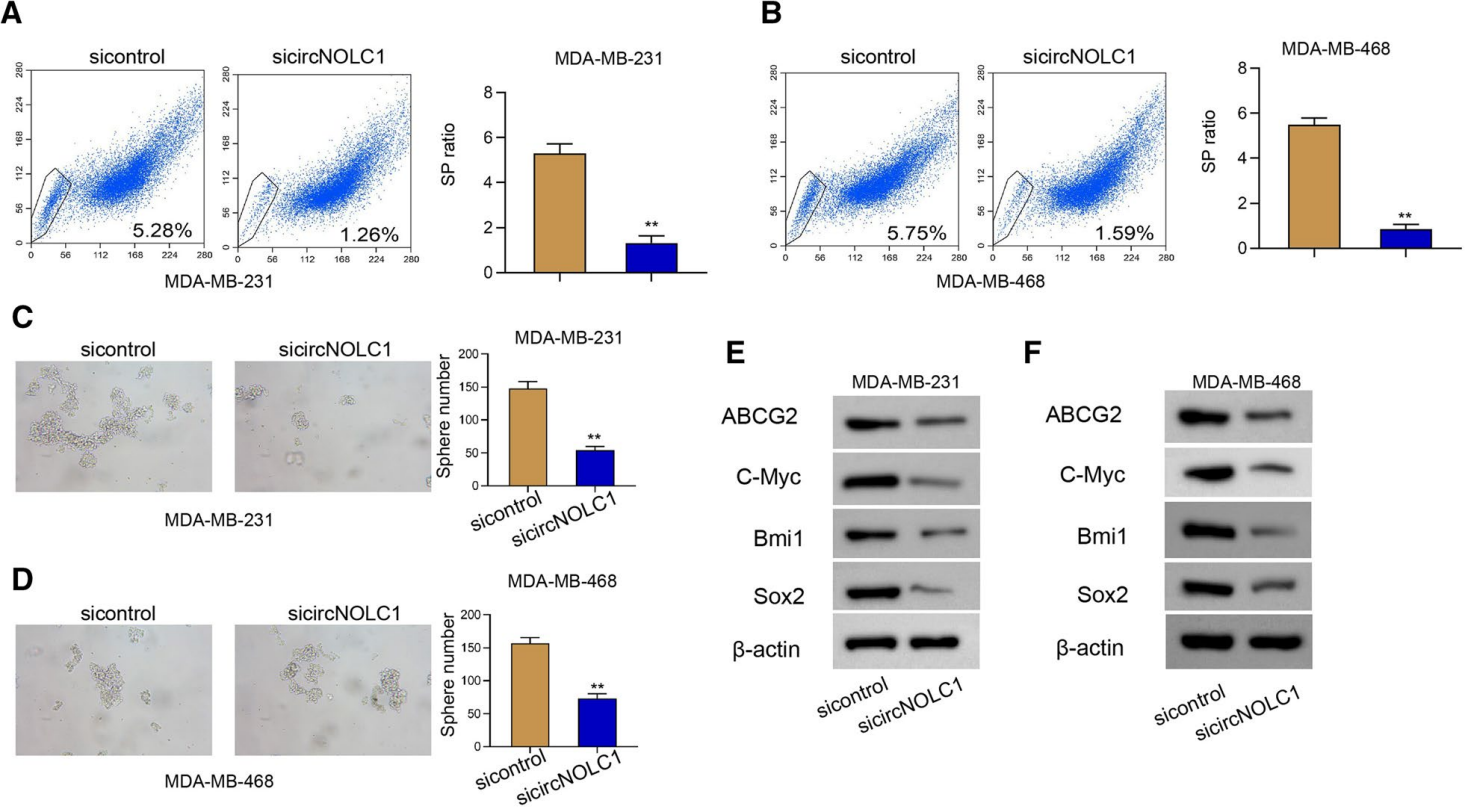
The inhibition of circNOLC1 attenuates cancer stem cell activity of breast cancer. **A**–**F** The MDA-MB-231 and MDA-MB-468 cells were treated with circNOLC1 shRNAs. **A** and **B** The SP ration was identified by Hoechst 33342 staining using flow cytometry analysis. **C** and **D** The sphere formation ability was determined by sphere formation assays. **E** and **F** The protein levels of ABCG2, C-Myc, Bmi1, Sox2 were detected by Western blot analysis. **P < 0.01

Next, we further determined the effect of circNOLC1 on cancer stem cell property of breast cancer. We observed that the inhibition of circNOLC1 decreased the SP ratio in MDA-MB-231 and MDA-MB-468 cells (Fig. 3A and B). Meanwhile, the sphere formation ability of MDA-MB-231 and MDA-MB-468 cells was attenuated by the silencing of circNOLC1 (Fig. 3C and D). The levels of ABCG2, C-Myc, Bmi1, and Sox2 were repressed by the depletion of circNOLC1 in MDA-MB-231 and MDA-MB-468 cells (Fig. 3E and F), implying that the inhibition of circNOLC1 attenuates cancer stem cell properties.

### CircNOLC1 function as a ceRNA for miR-365a-3p

**Fig. 4:**
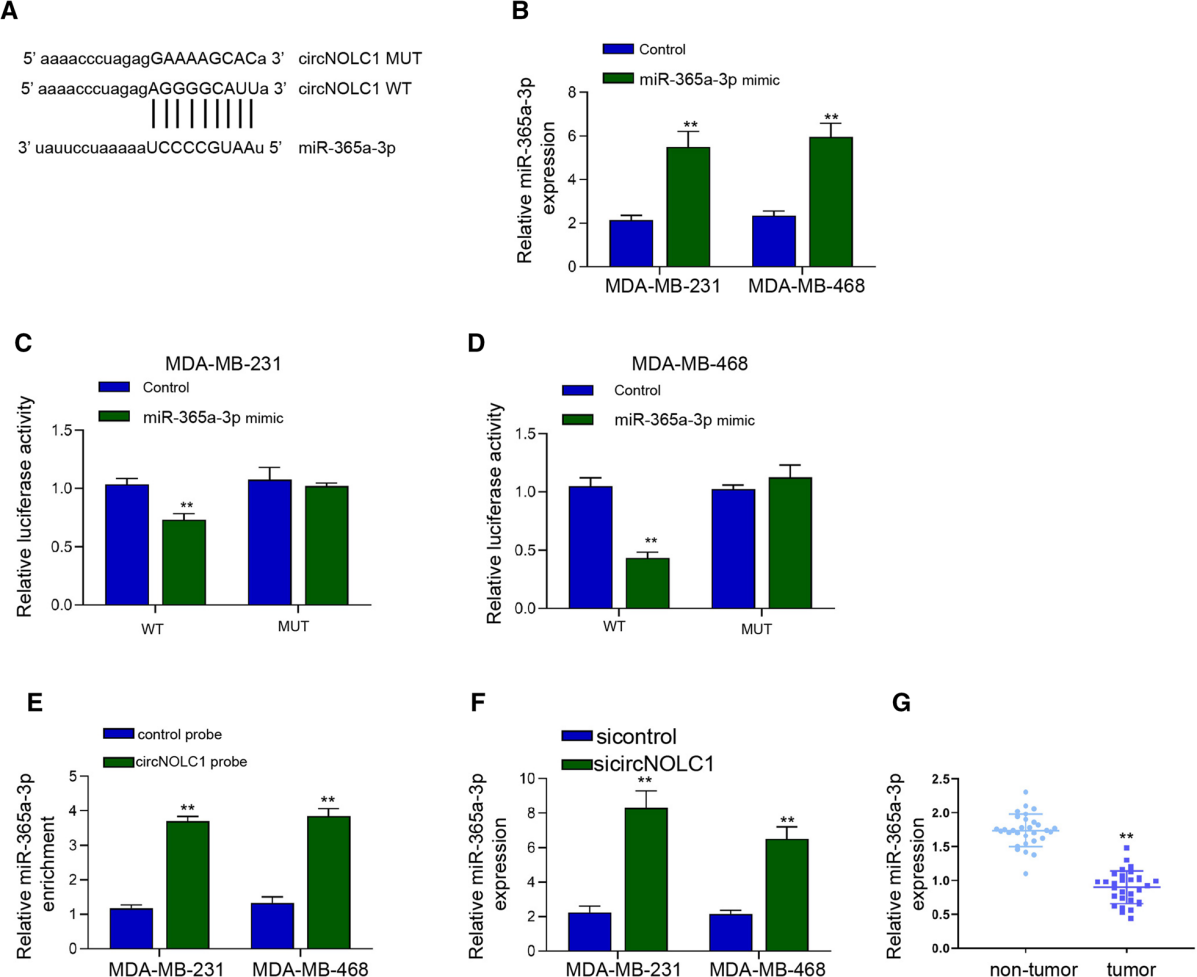
CircNOLC1 function as a ceRNA for miR-365a-3p. **A** The predicted binding sites of circNOLC1 and miR-365a-3p. **B**–**D** The MDA-MB-231 and MDA-MB-468 cells were treated with miR-365a-3p mimic. **B** The levels of miR-365a-3p were validated by qPCR. **C** and **D** The luciferase activities of circNOLC1 were determined by luciferase reporter gene assays. **E** The interaction between circNOLC1 and miR-365a-3p was confirmed using RNA pull down analysis. **F** The MDA-MB-231 and MDA-MB-231 cells were treated with circNOLC1 shRNAs. The levels of miR-365a-3p were tested by qPCR. **G** The levels of miR-365a-3p were examined by qPCR in clinical breast cancer tissues (n = 30). **P < 0.01

We then determined the potential mechanisms of circNOLC1 modulating breast cancer cells functions. The bioinformatics analysis revealed the potential binding sites between circNOLC1 and miR-365a-3p (Fig. 4A). The effectiveness of miR-365a-3p was validated in MDA-MB-231 and MDA-MB-468 cells (Fig. 4B). The luciferase activities of circNOLC1 were reduced in MDA-MB-231 and MDA-MB-468 cells after the treatment of miR-365a-3p mimic (Fig. 4C and D). RNA pull down assays showed that circNOLC1 was able to directly interact with miR-365a-3p in MDA-MB-231 and MDA-MB-468 cells (Fig. 4E). The inhibition of circNOLC1 enhanced miR-365a-3p levels in MDA-MB-231 and MDA-MB-468 cells (Fig. 4F). We observed that the expression of miR-365a-3p was repressed in breast cancer tissues compared with the non-tumor tissues (Fig. 4G).

### The inhibition of miR-365a-3p rescues circNOLC1 depletion-repressed proliferation and cancer stem cell activity of breast cancer

**Fig. 5:**
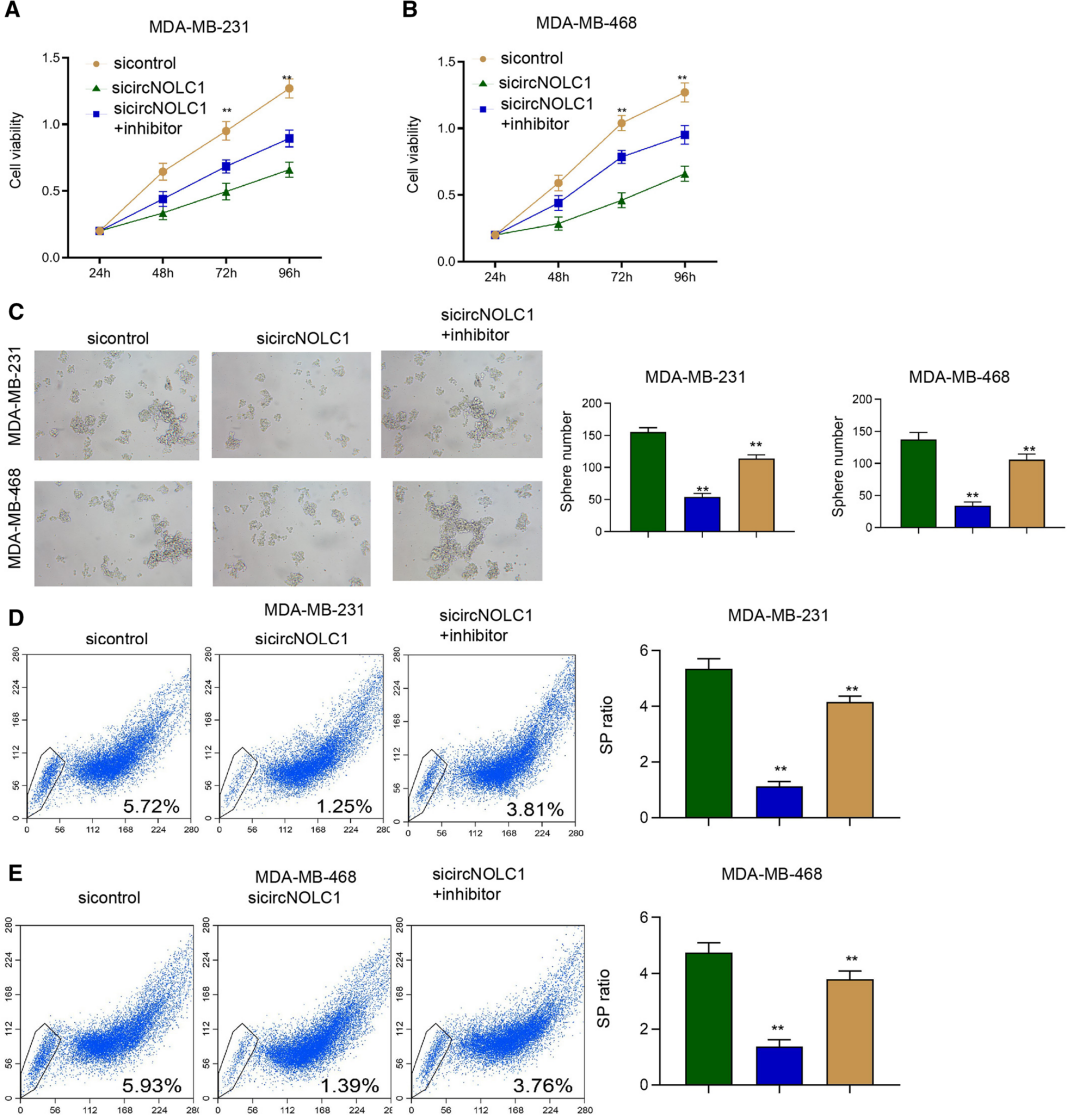
The inhibition of miR-365a-3p rescues circNOLC1 depletion-repressed proliferation and cancer stem cell activity of breast cancer. **A**–**E** The MDA-MB-231 and MDA-MB-468 cells were treated with circNOLC1 shRNAs with or without miR-365a-3p inhibitor. **A** and **B** The cell viability was detected by CCK-8 assays. **C** The sphere formation ability was determined by sphere formation assays. **D** and **E** The SP ration was identified by Hoechst 33342 staining using flow cytometry analysis. **P < 0.01

We then verified the function of circNOLC1/miR-365a-3p axis in the regulation of breast cancer functions. We observed that the silencing of circNOLC1 repressed the cell viability of MDA-MB-231 and MDA-MB-468 cells and the miR-365a-3p inhibitor rescued the viability (Fig. 5A and B). The inhibition of circNOLC1 suppressed sphere formation ability of MDA-MB-231 and MDA-MB-468 cells but the miR-365a-3p depletion restored the phenotype (Fig. 5C). Similarly, the knockdown of circNOLC1 inhibited the SP ratio in MDA-MB-231 and MDA-MB-468 cells, while the miR-365a-3p inhibitor reversed the inhibition (Fig. 5D and E), implying that the inhibition of miR-365a-3p rescues circNOLC1 depletion-repressed proliferation and cancer stem cell activity of breast cancer.

### MiR-365a-3p targets STAT3 in breast cancer cells

**Fig. 6:**
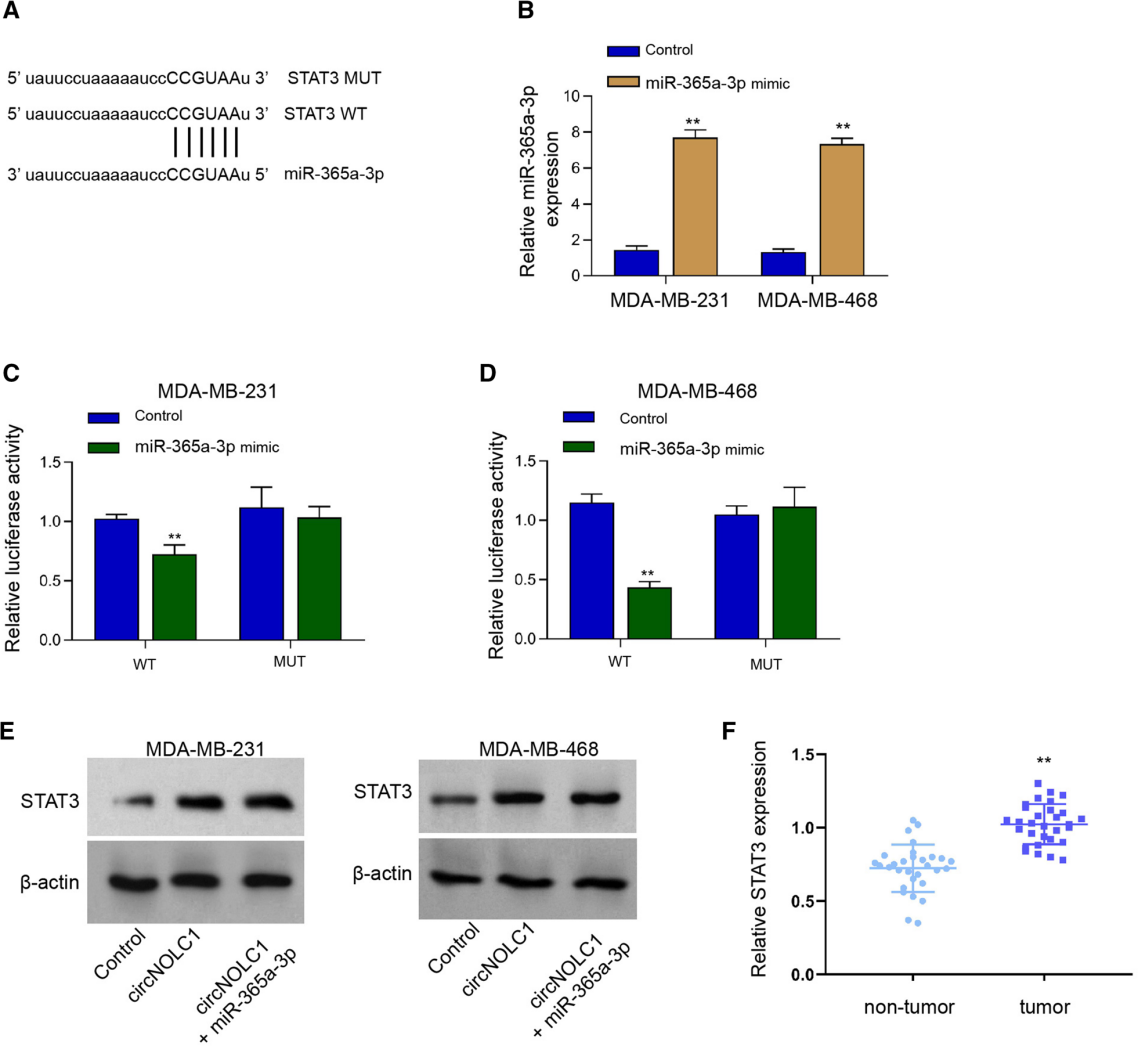
MiR-365a-3p targets STAT3 in breast cancer cells. **A** The predicted binding sites of STAT3 and miR-365a-3p. **B**–**D** The MDA-MB-231 and MDA-MB-468 cells were treated with miR-365a-3p mimic. **B** The levels of miR-365a-3p were validated by qPCR. **C** and **D** The luciferase activities of STAT3 were determined by luciferase reporter gene assays. **E** The MDA-MB-231 and MDA-MB-468 cells were treated with circNOLC1 overexpression plasmid and miR-365a-3p mimic. The protein levels of STAT3 were determined by Western blot analysis. **F** The levels of STAT3 were examined by qPCR in clinical breast cancer tissues (n = 30). **P < 0.01

We then identified a binding site between STAT3 and miR-365a-3p using bioinformatics analysis (Fig. 6A). The efficiency of miR-365a-3p mimic was confirmed in MDA-MB-231 and MDA-MB-468 cells (Fig. 6B). The luciferase activities of STAT3 were decreased in MDA-MB-231 and MDA-MB-468 cells under the treatment of miR-365a-3p mimic (Fig. 6C and D). The levels of STAT3 were induced by the overexpression of circNOLC1 while the treatment of miR-365a-3p mimic blocked the induction in MDA-MB-231 and MDA-MB-468 cells (Fig. 6E). We observed that the expression of STAT3 was enhanced in breast cancer tissues compared with the non-tumor tissues (Fig. 6F).

### The overexpression of STAT3 rescues miR-365a-3p-inhibited proliferation and cancer stem cell activity of breast cancer

**Fig. 7:**
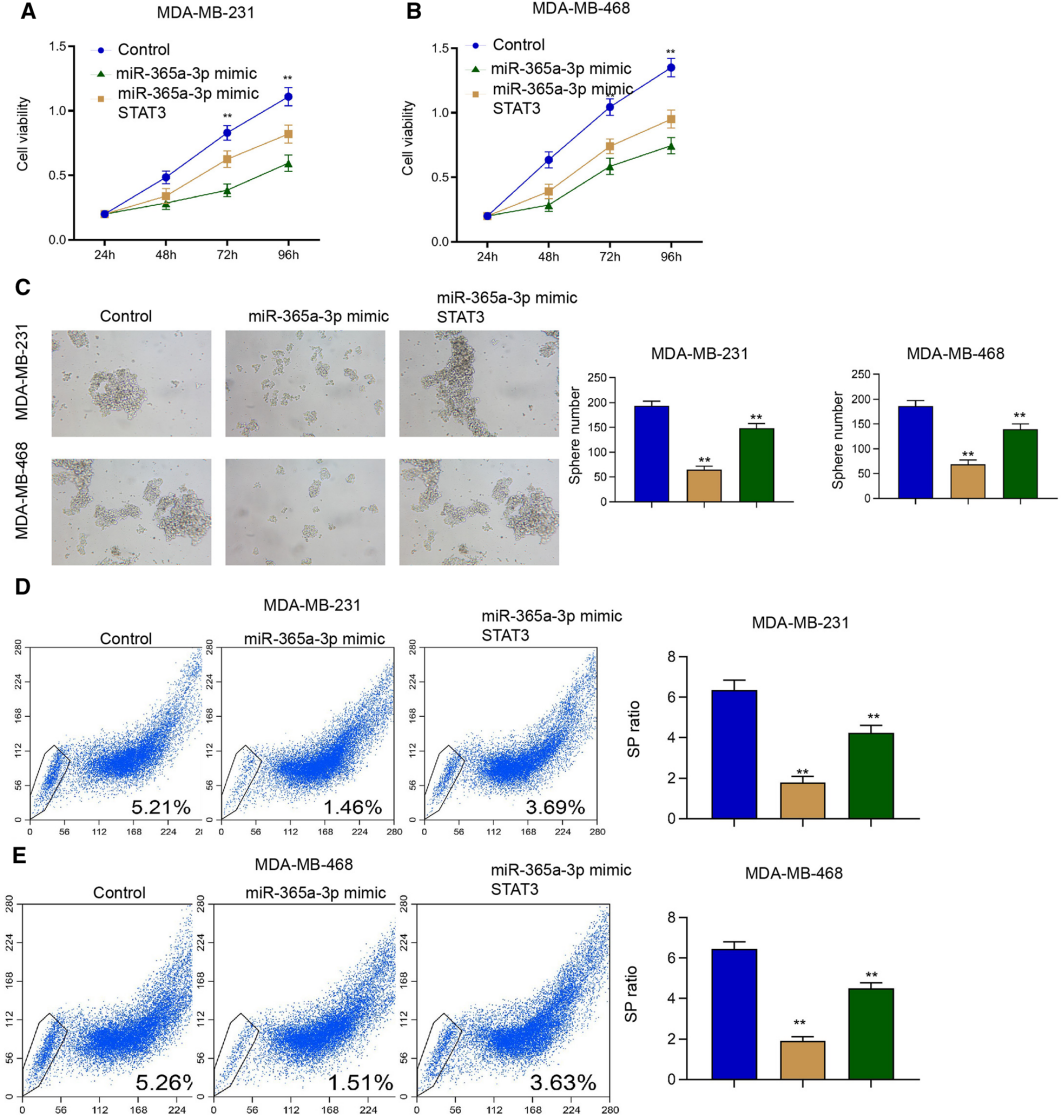
The overexpression of STAT3 rescues miR-365a-3p-inhibited proliferation and cancer stem cell activity of breast cancer. **A**–**E** The MDA-MB-231 and MDA-MB-468 cells were treated with miR-365a-3p mimic with or without STAT3 overexpressing plasmid. **A** and **B** The cell viability was detected by CCK-8 assays. **C** The sphere formation ability was determined by sphere formation assays. **D** and **E** The SP ration was identified by Hoechst 33342 staining using flow cytometry analysis. **P < 0.01

We then assessed the function of miR-365a-3p/STAT3 axis in the regulation of breast cancer functions. We observed that the miR-365a-3p mimic inhibited the cell viability of MDA-MB-231 and MDA-MB-468 cells and STAT3 overexpression rescued the viability (Fig. 7A and B). The miR-365a-3p mimic repressed sphere formation ability of MDA-MB-231 and MDA-MB-468 cells but the STAT3 overexpression restored the phenotype (Fig. 7C). Meanwhile, miR-365a-3p mimic reduced the SP ratio in MDA-MB-231 and MDA-MB-468 cells, while the STAT3 overexpression reversed the inhibition (Fig. 7D and E), implying that the overexpression of STAT3 rescues miR-365a-3p-inhibited proliferation and cancer stem cell activity of breast cancer.

### The overexpression of STAT3 rescues circNOLC1 depletion-attenuated proliferation and cancer stem cell activity of breast cancer

**Fig. 8:**
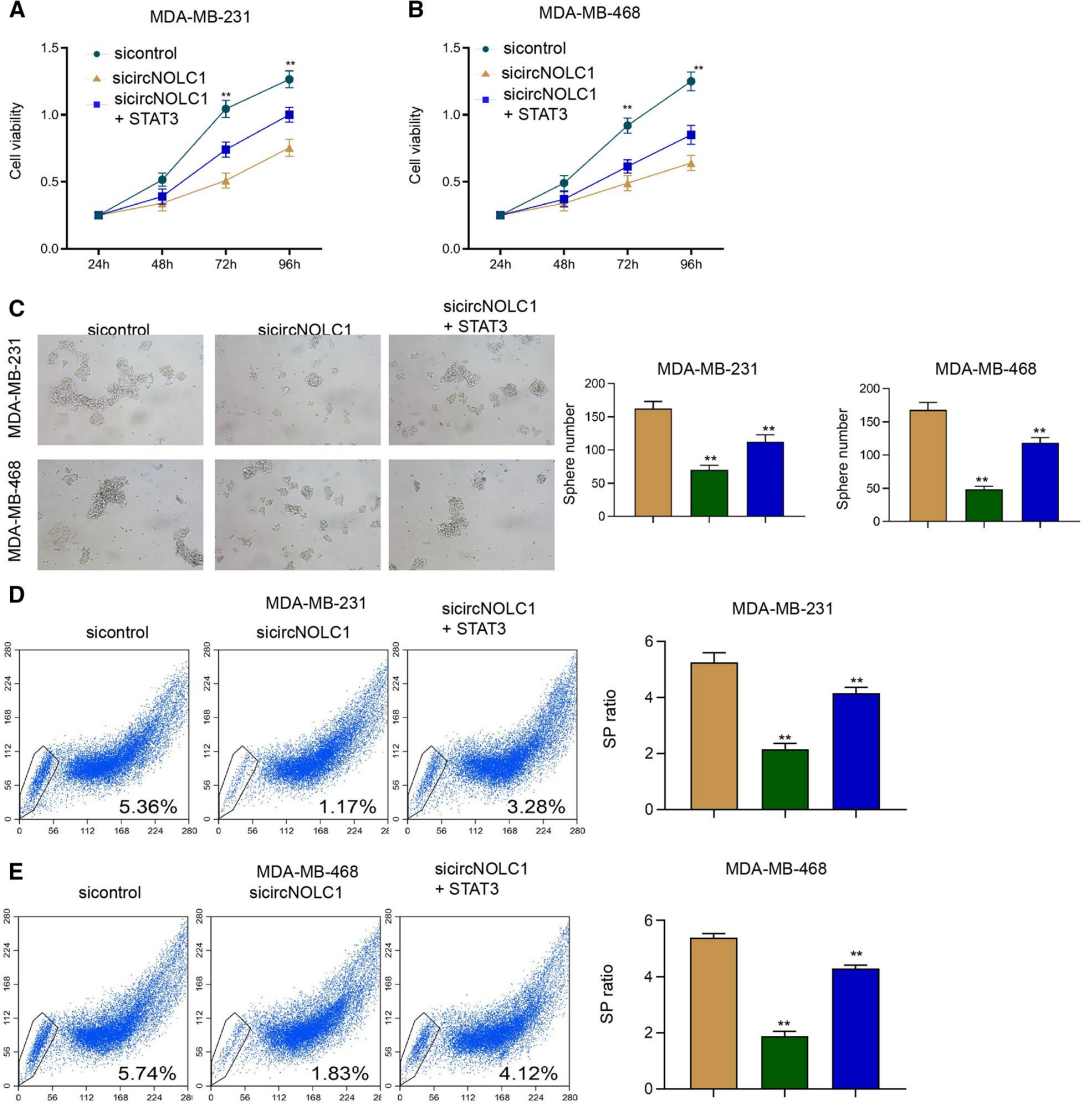
The overexpression of STAT3 rescues circNOLC1 depletion-attenuated proliferation and cancer stem cell activity of breast cancer. **A**–**E** The MDA-MB-231 and MDA-MB-468 cells were treated with circNOLC1 shRNAs with or without STAT3 overexpressing plasmid. **A** and **B** The cell viability was detected by CCK-8 assays. **C** The sphere formation ability was determined by sphere formation assays. **D** and **E** The SP ration was identified by Hoechst 33342 staining using flow cytometry analysis. **P < 0.01

We then analyzed the function of circNOLC1/STAT3 axis in the regulation of breast cancer functions. We observed that the silencing of circNOLC1 repressed the cell viability of MDA-MB-231 and MDA-MB-468 cells and the overexpression of STAT3 rescued the viability (Fig. 8A and B). The inhibition of circNOLC1 suppressed sphere formation ability of MDA-MB-231 and MDA-MB-468 cells but the STAT3 overexpression restored the phenotype (Fig. 8C). Similarly, the knockdown of circNOLC1 inhibited the SP ratio in MDA-MB-231 and MDA-MB-468 cells, while the STAT3 overexpression reversed the inhibition (Fig. 8D and E), implying that the overexpression of STAT3 rescues circNOLC1 depletion-attenuated proliferation and cancer stem cell activity of breast cancer.

### Propofol represses circNOLC1 expression by targeting STAT3

**Fig. 9:**
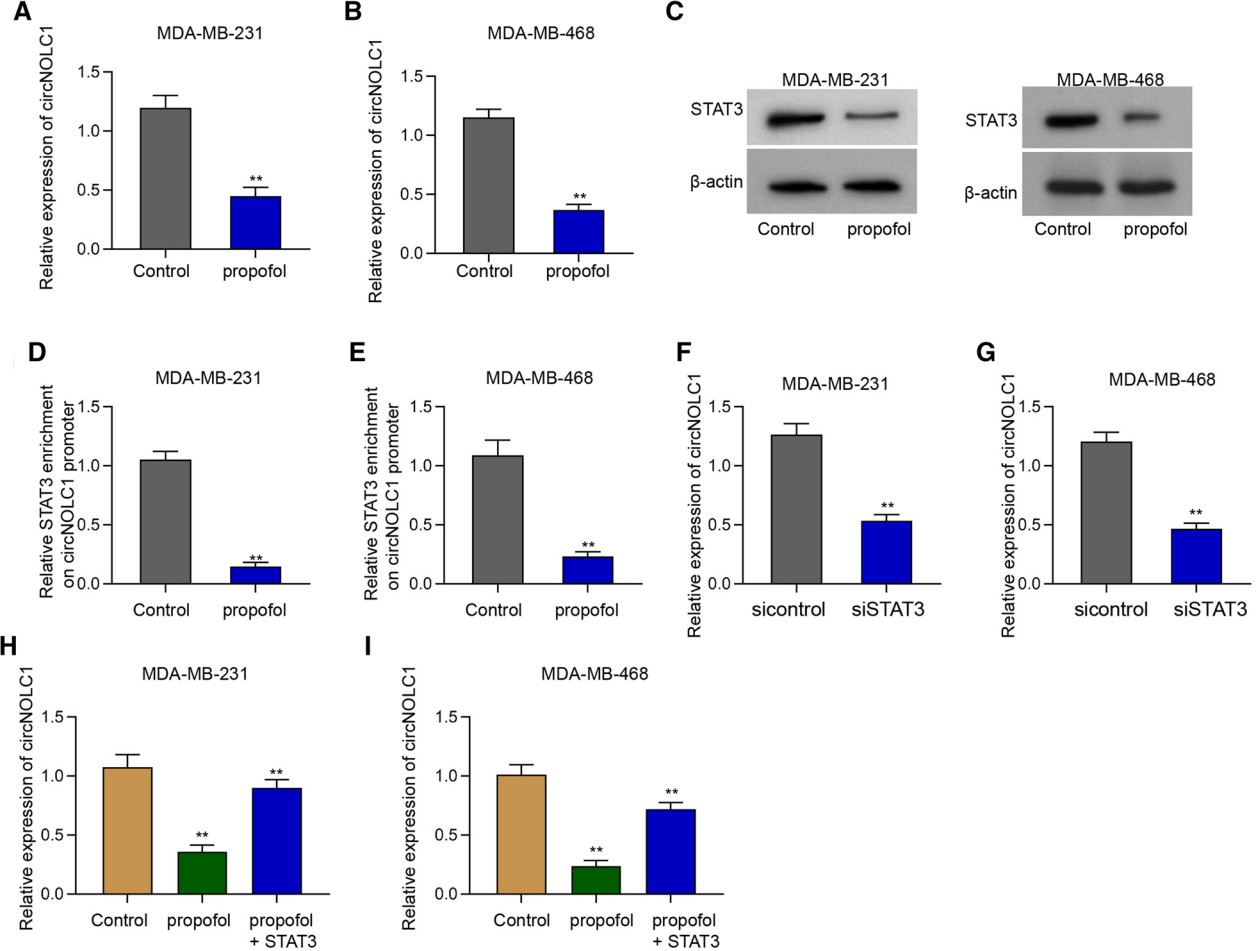
Propofol represses circNOLC1 expression by targeting STAT3. **A**–**E** The MDA-MB-231 and MDA-MB-468 cells were treated with propofol. **A** and **B** The expression of circNOLC1 was determined by qPCR. **C** The levels of STAT3 were detected by Western blot analysis. **D** and **E** The enrichment of STAT3 on circNOLC1 promoter was measured by ChIP assays. **F** and **G** The MDA-MB-231 and MDA-MB-468 cells were treated with STAT3 siRNA. The expression of circNOLC1 was determined by qPCR. **H** and **I** The MDA-MB-231 and MDA-MB-231 cells were treated with propofol with or without STAT3 overexpressing plasmid. The expression of circNOLC1 was determined by qPCR. **P < 0.01

We then tried to explore the inhibitor of circNOLC1 in the treatment of breast cancer cells. We identified that the expression of circNOLC1 and STAT3 was repressed by the treatment of propofol in MDA-MB-231 and MDA-MB-468 cells (Fig. 9A–C). The enrichment of STAT3 on circNOLC1 promoter was inhibited by propofol in MDA-MB-231 and MDA-MB-468 cells (Fig. 9D and E), implying that propofol is able to reduce circNOLC1 expressing by inhibiting STAT3 recruitment on circNOLC1 promoter. We validated that the expression of circNOLC1 was suppressed by the silencing of STAT3 in MDA-MB-231 and MDA-MB-468 cells (Fig. 9F and G). The inhibition of circNOLC1 expression by propofol was rescued under the co-treatment of STAT3 overexpression in MDA-MB-231 and MDA-MB-468 cells (Fig. 9H and I).

### The overexpression of circNOLC1 rescues propofol-attenuated proliferation and cancer stem cell activity of breast cancer

**Fig. 10:**
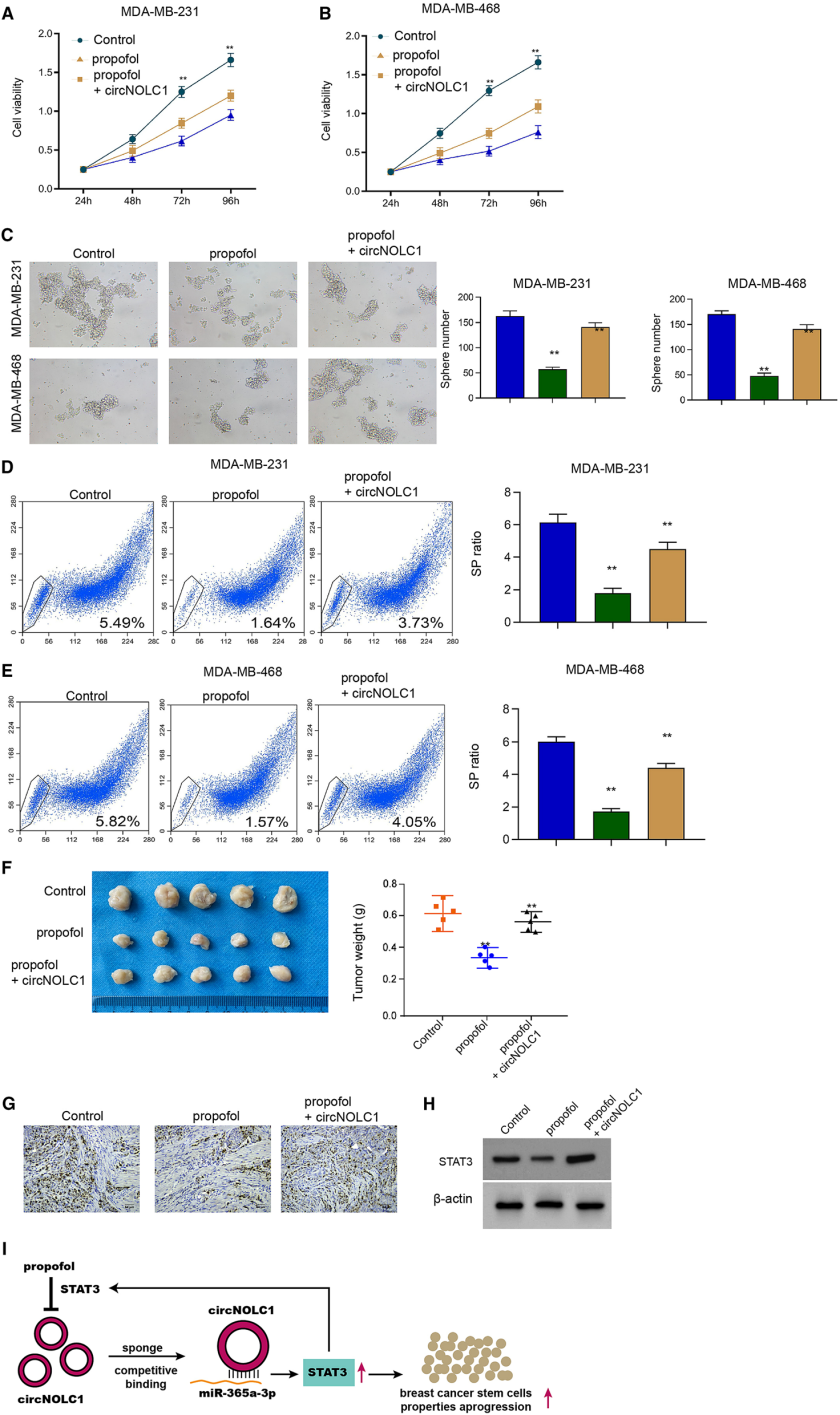
The overexpression of circNOLC1 rescues propofol-attenuated proliferation and cancer stem cell activity of breast cancer*.* **A–E** The MDA-MB-231 and MDA-MB-468 cells were treated with propofol with or without circNOLC1 overexpressing plasmid. **A** and **B** The cell viability was detected by CCK-8 assays. **C** The sphere formation ability was determined by sphere formation assays. **D** and **E** The SP ration was identified by Hoechst 33342 staining using flow cytometry analysis. **F**–**H** The effect of propofol/circNOLC1 axis on tumor growth of MDA-MB-231 cells was analyzed by tumorigenicity analysis in nude mice. The tumor weight (**F**), Ki-67 levels (**G**), and STAT3 levels were shown. **I** A model of this study was shown, in which circNOLC1 contributed to CSCs properties and progression of breast cancer by targeting miR-365a-3p /STAT3 axis and propofol inhibited circNOLC1 by repressing STAT3 in a feedback mechanism. **P < 0.01

Functionally, we found that the treatment of propofol repressed the cell viability of MDA-MB-231 and MDA-MB-468 cells and the overexpression of circNOLC1 rescued the viability (Fig. 10A and B). Propofol suppressed sphere formation ability of MDA-MB-231 and MDA-MB-468 cells but the circNOLC1 overexpression restored the phenotype (Fig. 10C). Similarly, propofol inhibited the SP ratio in MDA-MB-231 and MDA-MB-468 cells, while the circNOLC1 overexpression reversed the inhibition (Fig. 10D and E). Moreover, tumorigenicity analysis showed that the treatment of propofol attenuated the tumor growth of MDA-MB-231 cells, while the overexpression of circNOLC1 could reverese the effect of propofol in the model (Fig. 10F–H).

## Discussion

Breast cancer is a dreadful female malignancy globally and CSCs play crucial functions in breast cancer development. CircRNAs have drawn great attention in cancer research area and propofol is a widely applied intravenous anesthetic agent, that was also implied to affect the progression of breast cancer. In the present study, we uncovered the oncogenic function of circNOLC1 in CSCs of breast cancer and the inhibitory impact of propofol on circNOLC1.

Previous studies have reported the crucial roles of circRNAs in the modulation of CSCs of breast cancer. For example, circRGPD6 inhibits cancer stem cell-regulated metastasis of breast cancer by the miR-26b/YAF2 signaling [24]. Meanwhile, circNOLC1 is enhanced by NF-kB, and induces prostate cancer development by miR-647/PAQR4 axis [15]. CircNOLC1 enhances progression and tumorigenesis of epithelial ovarian cancer by interacting ESRP1 and regulating RhoA and CDK1 expression [25]. Our data showed that the expression of circNOLC1 was induced in breast cancer tissues compared with the non-tumor tissues. The silencing of circNOLC1 was able to repress the proliferation of breast cancer cells in vivo and in vitro. The inhibition of circNOLC1 reduced the invasion and migration ability of breast cancer cells. The mRNA and protein levels of E-cadherin were enhanced but Vimentin levels were reduced by the silencing of circNOLC1. The depletion of circNOLC1 inhibited CSCs properties in breast cancer cells. These data indicate the innovative impact on the induction of CSCs and progression of breast cancer, validating the essential function of circRNAs in breast cancer cells.

Moreover, it has been reported that miR-365a-3p reduces c-Rel-regulated NF-κB signaling during pancreatic cancer progression [17]. Long Non-Coding RNA NEAT1 sponges miR-365a-3p to enhance progression of gastric cancer by tarheting ABCC4 [19]. MiR-365a-3p inhibits metastasis and growth of colorectal cancer by targeting ADAM10/JAK/STAT signaling [26]. MT1DP loaded by folate-modified liposomes modulates erastin-stimulated ferroptosis of non-small cell lung cancer cells by modulating miR-365a-3p/NRF2 axis [27]. Our mechanical data showed that circNOLC1 functioned as a ceRNAs for miR-365a-3p and the inhibition of miR-365a-3p rescued circNOLC1 depletion-repressed proliferation and cancer stem cell activity of breast cancer. MiR-365a-3p targeted STAT3 in breast cancer cells and circNOLC1 enhanced STAT3 expression by sponging miR-365a-3p. The overexpression of STAT3 could reverse miR-365a-3p or circNOLC1 depletion-inhibited proliferation and cancer stem cell properties of breast cancer. These data indicate a downstream molecular mechanism by which circNOLC1 contributes to CSCs of breast cancer via targeting miR-365a-3p/STAT3 axis. The clinical correlation of circNOLC1/miR-365a-3p/STAT3 axis and other targets of circNOLC1 and miR-365a-3p in the regulation of breast cancer should be explored by more investigations. Propofol have presented anti-tumor function in breast cancer by multiple mechanisms. Propofol regulates cytotoxic T lymphocyte, natural killer cell, and cancer cell function in breast cancer patients [28]. Propofol enhances breast cancer cell apoptosis via downregulating miR-24 signaling [29]. Propofol epigenetically modulates trastuzumab resistance of breast cancer via IL-6/miR-149-5p signaling [30]. In addition, propofol attenuates metastasis of colon cancer by STAT3/HOTAIR axis through the activation of WIF-1 and the suppression of Wnt signaling [31]. Interestingly, we found that propofol repressed circNOLC1 expression by targeting STAT3 in a feedback loop. These data not only uncover the inhibitory effect of propofol on circNOLC1, but also provide new mechanisms of propofol in repressing breast cancer. In this study, we observed the similar results in MDA-MB-231 and MDA-MB-468 cells and the results should be validated in more cell lines in the future. Meanwhile, there were still some limitations of this study. For example, the signaling pathway mediated by STAT3 in propofol/circNOLC1/miR-365a-3p/STAT3 axis should be explore in future investigations.

## Conclusions

In conclusion, we concluded that circNOLC1 contributed to CSCs properties and progression of breast cancer by targeting miR-365a-3p /STAT3 axis and propofol inhibited circNOLC1 by repressing STAT3 in a feedback mechanism (Fig. 10I).

## Data Availability

The datasets used and analysed during the current study are available from the corresponding author on reasonable request.
